# Molecular stratifications, biomarker candidates and new therapeutic options in current medulloblastoma treatment approaches

**DOI:** 10.1007/s10555-020-09854-1

**Published:** 2020-01-22

**Authors:** Otília Menyhárt, Balázs Győrffy

**Affiliations:** 1 2nd Department of Pediatrics and Department of Bioinformatics, Semmelweis University, Budapest, Hungary; 2 Research Centre for Natural Sciences, Cancer Biomarker Research Group, Institute of Enzymology, Magyar tudósok körútja 2, Budapest, H-1117 Hungary

**Keywords:** Prognostic biomarker, Risk stratification, Clinical trial, WNT, SHH, Group 3, Group 4, Immunotherapy

## Abstract

Medulloblastoma (MB) is the most common malignant childhood tumor of the brain. Multimodal treatment consisting of surgery, radiation therapy, and chemotherapy reduced cumulative incidence of late mortality but increased the incidence of subsequent neoplasms and severe, incapacitating chronic health conditions. Present treatment strategies fail to recognize heterogeneity within patients despite wide divergence in individual responses. The persistent mortality rates and serious side effects of non-targeted cytotoxic therapies indicate a need for more refined therapeutic approaches. Advanced genomic research has led to the accumulation of an enormous amount of genetic information and resulted in a consensus distinguishing four molecular subgroups, WNT-activated, SHH-activated, and Group 3 and 4 medulloblastomas. These have distinct origin, demographics, molecular alterations, and clinical outcomes. Although subgroup affiliation does not predict response to therapy, new subgroup-specific markers of prognosis can enable a more layered risk stratification with additional subtypes within each primary subgroup. Here, we summarize subgroup-specific genetic alterations and their utility in current treatment strategies. The transition toward molecularly targeted interventions for newly diagnosed MBs remains slow, and prospective trials are needed to confirm stratifications based on molecular alterations. At the same time, numerous studies focus at fine-tuning the intensity of invasive radio- and chemotherapies to reduce intervention-related long-term morbidity. There are an increasing number of immunotherapy-based treatment strategies including immune checkpoint-inhibitors, oncolytic viruses, CAR-T therapy, and NK cells in recurrent and refractory MBs. Although most trials are in early phase, there is hope for therapeutic breakthroughs for advanced MBs within the next decade.

## Introduction

Medulloblastoma (MB) is the most common malignant childhood tumor of the brain, accounting for ~ 20% of all CNS tumors among children [1]. Current multimodal treatment consisting of surgery, radiation therapy, and chemotherapy allows 60–80% long-term survival [2]. According to a report from the Childhood Cancer Survivor Study comparing long-term mortality and morbidity data, cumulative incidence of all-cause late mortality became significantly lower for MB patients diagnosed in the 1990s compared to the 1970s [3]. Still, more than one-third of patients die within 5 years after diagnosis [1], and the median survival of relapsed/refractory MB is less than 1 year [4]. Each treatment component is responsible for late complications leading to severe, debilitating chronic health conditions [3]. Late mortality remains a significant problem, largely attributable to disease recurrence and secondary malignancies [5]. The current clinical and pathological feature-based risk stratification fails to consider heterogeneity within standard- and high-risk patients. The wide disparities among patient outcomes, the deteriorating quality of life expectations for survivors, and the failure of aggressive, high intensity multimodal therapies to extend life expectancy of patients with recurrent disease illuminate the need for new, more effective treatment approaches.

Integration of molecular data provided by advanced genomics started an exciting refinement of MB classifications [6]: the current consensus agrees upon four distinct molecular subgroups, wingless-activated (WNT-MB), sonic-hedgehog-activated (SHH-MB), and Group 3 and Group 4 MBs [7], each characterized by distinct variety of genetic alterations, transcriptional/methylation profiles, and clinical outcomes [2, 8–10]. Subgroup identity is linked with markedly different survival, although subgroup affiliation does not correlate with response to therapy [11]. Within each primary subgroup, additional molecular markers started to emerge, leading to more refined risk stratifications [2]. Ongoing trials explore personalized treatment strategies for patients harboring specific genetic aberrations.

In this review, we summarize treatment approaches, outline the evolving landscape of molecular classifications, and discuss markers with prognostic relevance. Our main focus is the ongoing transition toward molecular-based risk-adapted strategies in the clinic. We review currently recruiting clinical trials available for both newly diagnosed and recurrent/refractory MB patients. A decent tendency among current trials is the increasing number of targeted and immunotherapy-based approaches, although up till now exclusively for patients with recurrent/refractory MBs.

### Clinical classification and current treatment protocols

Current treatment approaches consider two main factors for the selection of postoperative therapy: risk of treatment toxicity and risk of recurrence. Risk of treatment toxicity is especially significant for infants and children younger than 3 years of age. Risk of recurrence is considered to be high in case of disseminated or metastatic disease or suboptimal resection, with a tumor residue ≥ 1.5cm^2^. Certain histologic subtypes, such as anaplastic and large cell tumors, have also been associated with poor outcome [12]. Three treatment cohorts can be separated based on these criteria: children older than 3 years of age with average-risk disease, children older than 3 years of age with high-risk disease, and infants and children younger than 3 years of age.

#### Surgical intervention

Surgical goal is maximal safe resection that alleviates increased intracranial pressure and removes as much tumor tissue as possible without causing adverse neurological sequelae, such as cranial nerve deficits or persistent ataxia. Posterior fossa syndrome (cerebellar mutism) still occurs in about one-quarter of patients with MB resection. Development of modern imaging and surgical techniques prompted gross total or near-total resection in most patients [13]. Meanwhile, the magnitude of the extent of resection may not be as significant as earlier thought to be: an inconclusive retrospective systematic review on the extent of resection and disease outcome warrants further evaluation [14]. Prognostic benefits may also be attenuated by molecular subgroups: gross total resection in Group 4 MB patients conferred progression-free survival benefit compared to subtotal resection, especially in the presence of disease dissemination, while the extent of resection was not linked to overall survival (OS) [13].

#### Radiation therapy (RT)

Following surgery patients are treated with external beam irradiation to the craniospinal axis with an additional boost to the tumor site, with varying radiation doses across risk groups. Most recurrences (50–70%) are local; therefore treatment is limited to the tumor bed compared to the entire posterior fossa which spares critical brain structures [15]. RT is usually initiated 30 days after surgery, since delayed RT administration is associated with lower survival [8, 16]. At the same time, a more recent study identified decreased 5-year OS in patients with RT initiated within 3 weeks after surgery, indicating ample time for healing is also paramount [17].

Patients with average-risk disease are treated with 23.4 Gy craniospinal irradiation (CSI) to the entire brain and spine, with posterior fossa boost for a total dose of 54–55.8 Gy [18–22]. In contrast, high-risk patients receive 36–39.6 Gy CSI with an additional boost to the tumor site, for a total dose of 54–55.8 Gy [21]. Such RT doses are associated with neurocognitive impairment, affecting younger children more negatively [23]. However, reduced-dose neuraxis photon RT results in increased frequency of relapse and decreased survival [24]. In the phase III Children’s Oncology Group ACNS0331 trial**,** 5.4 Gy reduction in the CSI dose in children between 3 and 7 years of age produced inferior survival compared to standard**-**dose RT [25]. Deferral of postoperative RT even in the modern era of chemotherapy is associated with poorer OS and is therefore not recommended for children older than 3 years of age [26].

Newer techniques, such as proton radiation therapy and intensity-modulated RT utilized for the posterior fossa boost and spine RT, limit irradiation of the normal tissues with acceptable toxicity and survival similar to conventional RT [27]. Reduced normal tissue dose also relieves post-radiation ototoxicity, leading to preserved hearing and improved quality of life [28, 29]. Nevertheless, additional studies are needed to determine if proton beam RT provides clinically meaningful long-term cognitive sparing compared to photon beam RT protocols [30].

#### Chemotherapy

Following postsurgical RT, chemotherapy became accepted as a standard treatment as it improves survival and reduces RT-related adverse effects, especially in younger children. Numerous risk-adapted multimodal treatment protocols have been tested in clinical trials. A randomized, multicenter trial (HIT’91) compared the long-term effects of a neoadjuvant (“sandwich”) chemotherapy (incorporating ifosfamide, etoposide, high-dose methotrexate, cisplatin, and cytarabine, followed by CSI and maintenance chemotherapy) versus “maintenance” chemotherapy after radiation (consisting of vincristine complementary to RT and eight cycles with CCNU, vincristine and cisplatin 6 weeks after RT), adapted from the “Packer protocol.” The “maintenance” regimen resulted in significantly higher 10-year OS compared to the “sandwich” approach among patients with M0 (91% vs. 62%) or M1 (70% *vs.* 34%) disease [31, 32].

The phase III Children’s Oncology Group (COG) study adopted a weekly vincristine treatment during RT, followed by eight cycles of chemotherapy including vincristine and cisplatin plus CCNU, or vincristine and cisplatin plus cyclophosphamide. The strategy leads to 76% and 81% 10-year event-free and overall survival, respectively, in average-risk patients, with no differences in efficacy between the two chemotherapy regimens [22, 33], producing at least as good outcome as previous, more intensive treatment protocols.

The current treatment approach includes risk-adapted RT and four cycles of cyclophosphamide-based, dose-intensive chemotherapy (incorporating cisplatin, vincristine, and cyclophosphamide), followed by stem cell or bone marrow rescue. This regimen produced 85% 5-year survival (95% CI 75–94) in the average-risk MB population and 70% 5-year survival (95% 54–84) in the high-risk population [21]. Histological subtype also correlated with 5-year event-free survival and was lowest (57%) for large cell anaplastic tumors [21].

Additional ongoing clinical trials test the feasibility of alternative treatment regimes in newly diagnosed MBs. The recruiting SIOP PNET 5 MB phase II/III trial (NCT02066220) evaluates the outcome in patients with non-WNT “standard-risk” biological profiles, defined by nuclear beta-catenin immune-negativity, after concurrent carboplatin and radiotherapy, followed by eight cycles of maintenance chemotherapy (Table 1).

**Table 1:** List of ongoing clinical trials currently recruiting newly diagnosed medulloblastoma patients

ID	Phase	Treatment	Treatment arms	Subgroup	Risk	Eligible age cohort	Allocation	Primary outcome measure	Estimated primary completion date
NCT01878617	II	Treatment stratification based on both clinical risk (low-, standard-, intermediate-, or high-risk) and molecular subtype (WNT, SHH, or non-WN/non-SHH)	Low-risk (stratum W1): reduced-dose craniospinal irradiation, lower dose of cyclophosphamide	WNT (strata W): positive for WNT biomarkers	All risk categories	3 years to 39 years	Non-randomized	Progression-free survival	2023
			atypical (W2): standard-dose craniospinal radiation, standard chemotherapy (cisplatin, vincristine, cyclophosphamide)					Change in VO2 peak values after aerobic training intervention; change in spatial span backward standard score	
			High-risk (W3): high does craniospinal radiation, standard chemotherapy						
			Standard-risk (S1): standard-dose craniospinal radiation, chemotherapy, vismodegib maintenance therapy for skeletally	SHH (strata S): positive for SHH biomarkers					
			High-risk (S2): high-dose craniospinal radiation, chemotherapy, vismodegib maintenance therapy for skeletally mature patients						
			Standard-risk (N1): standard-dose craniospinal radiation, chemotherapy	Non-WNT/non-SHH, failed, or indeterminate (Strata N): negative for WNT and SHH biomarkers for results are indeterminable				Progression-free survival	
			intermediate risk (N2): standard-dose craniospinal radiation, standard chemotherapy (cisplatin, vincristine, cyclophosphamide) for four cycles intermixed with an additional three cycles of chemotherapy with pemetrexed and gemcitabine					Change in VO2 peak values after aerobic training intervention; change in spatial span backward standard score	
			High-risk (N3): high-dose craniospinal radiation, standard chemotherapy with an additional three cycles of chemotherapy with pemetrexed and gemcitabine						
NCT01857453	II	Reduced irradiation compensated by chemotherapy	Carboplatin + etoposide-based chemotherapy followed by radiation therapy with 24 Gy on the in toto neuro axis and 54 Gy on the post-operative bed		Standard-risk	18 years to 70 years	Single group assignment	Survival without disease at 1 year	2018
NCT02025881	I/II	Sequential high-dose chemotherapy	Carboplatin + etoposide then thiotepa then cyclophosphamide + busilvex		High-risk	Up to 5 years	Single group assignment	Phase I- maximum tolerated dose; phase II- event- free survival up to 3 years	2018
NCT02875314	IV	Induction chemotherapy followed by either a single cycle of three tandem cycles	Single cycle (thiotepa, etoposide, and carboplatin for 6 days)	Non-WNT/non-SHH	High-risk	Up to 10 years	Randomized	Event-free survival, overall survival	2020
			three tandem cycles (thiotepa and carboplatin over 2 days, three cycles)						
NCT02724579	II	Reduced craniospinal radiotherapy	Reduced craniospinal radiotherapy (18 Gy) with a limited target volume boost to the tumor bed of 36 Gy for a total of 54 Gy and reduced chemotherapy (no vincristine during chemotherapy and reduced-dose maintenance chemotherapy)	WNT	Standard-risk	3 years to 21 years	Single group assignment	Progression-free survival	2025
NCT02066220	II/III	Risk-specific radio- and chemotherapy	Low-risk: radiotherapy and reduced-intensity maintenance chemotherapy consisting of 3 courses of cisplatin, CCNU, and vincristine alternating with three courses of cyclophosphamide and vincristine	ẞ-catenin nuclear immunopositivity (non-metastatic)	Low- and standard- risk	3 years to 21 years	randomized	3-year event-free survival	2024
			Standard-risk: radiotherapy with carboplatin or radiotherapy without carboplatin and maintenance chemotherapy consisting of four courses of cisplatin, CCNU, and vincristine alternating with four courses of cyclophosphamide and vincristine	ẞ-catenin nuclear immunonegativity (non-metastatic)					

Abbreviations: CSI, craniospinal radiation; PFS, progression-free survival; OS, overall survival; EFS, event-free survival; DFS, disease-free survival

Up to 30% of MB patients are diagnosed with a metastatic disease [34]. Despite high intensity RT and chemotherapy, the risk of recurrence and death remains elevated. A Children’s Oncology Group Phase I/II trial for metastatic patients administered 36 Gy CSI with boosts to the sites of disease, complemented with carboplatin along with vincristine during radiation, followed by six maintenance cycles of cyclophosphamide and vincristine with or without cisplatin. The regimen without cisplatin resulted in 82% 5-year survival, compared to 68% in the cisplatin arm [35]. Another approach for high-risk patients included a reduced-dose CSI (23.4 to 30.6 Gy) complemented with tandem high-dose chemotherapy (2 cycles pre- and 4 cycles post RT) and autologous stem cell transplantation. The treatment resulted in 70% 5-year event-free and 74% overall survival rates [36], although sample size was low and longer follow-up is needed to evaluate the potential long-term risks of high-dose chemotherapy, especially as chemotherapy may aggravate neurological adverse effects [37]. The HIT’2000 trial for patients with gross leptomeningeal dissemination (M2/3) adapted an intensified “sandwich”-regimen, including strengthened induction chemotherapy (comprising two cycles of intravenous cyclophosphamide, vincristine, methotrexate, carboplatin, etoposide, and concomitant intraventricular methotrexate), hyperfractionated CSI, and four cycles of maintenance chemotherapy (consisting of cisplatin, CCNU, and vincristine). The trial resulted in superior survival rates compared to the preceding HIT’91 trial: the 5-year OS reached 74% (95% CI 66–82%), with histology and nonresponse to the first chemotherapy cycle as independent risk factors [38]. Survival was associated with molecular subgroup identity and the presence of *MYCC/MYCN* amplifications, suggesting that these may be useful biomarkers in future treatment stratifications [38].

Infants and children under the age of 3 are preferably treated by surgery and chemotherapy alone due to high-risk of radiation-induced morbidity [39]. The CCG-99703 protocol consists of high-dose chemotherapy (vincristine, cyclophosphamide, etoposide, and cisplatin) followed by autologous hematopoietic cell rescue, while the HIT-SKK’92 protocol endorses systemic chemotherapy and intraventricular therapy (intravenous and intraventricular methotrexate, vincristine, cyclophosphamide, and carboplatin) [40, 41]. HIT-SKK’92 led to better outcomes in patients with gross total resection and without metastases compared to patients with residual or metastatic disease [41–43]. Delay of radiation therapy may be particularly favorable in young children with desmoplastic/extensive nodular histology [44], and sequential high-dose chemotherapy following the CCG-99703 protocol produced an excellent outcome for SHH-MB patients with classical histology [39].

Novel protocols are being evaluated to circumvent the debilitating complications linked to radiation therapy by extending the age range of young patients (Table 1). A phase II (NCT02025881) open-label, non-randomized trial focuses on children under the age of 5 with newly diagnosed high-risk MBs and investigates the efficacy of a sequential high-dose chemotherapy without radiotherapy with stem cell support. Risk assignment is based on histology (large cell/anaplastic (LCA) or other unfavorable), dissemination, and the presence of *MYC* and *MYCN* amplifications, and the chemotherapy regimen includes carboplatin + etoposide, then thiotepa, then cyclophosphamide, and busilvex. The study includes a phase I dose-finding part to determine the dose of cyclophosphamide that can be given in combination with busilvex. The phase IV HeadStart4 trial (NCT02875314) focuses on newly diagnosed high-risk children (up to 10 years of age) mainly consisting of non-WNT/non-SHH-MBs, and evaluates the efficacy of a high intensity induction therapy (including vincristine, cisplatin, cyclophosphamide, etoposide and high-dose methotrexate) followed by either a single cycle (carboplatin, thiotepa, etoposide) or three tandem cycles (carboplatin, thiotepa) of consolidation marrow-ablative chemotherapy with autologous hematopoietic progenitor cell rescue. Risk assessment is based on clinical features such as age (all MBs under the age of 6), histology, and stage (high disease stage with classic or LCA histology between the ages of 6 and 10).

In adults, MBs are rare, accounting for < 1% of all CNS-tumors, and most adult patients belong to the standard clinical risk group. Due to the relatively low incidence of adult MBs, there are no randomized clinical trials upon which to base treatment recommendations. After maximal safe resection, adult patients are treated with normofractionated CSI of 30–36 Gy followed by a tumor bed boost to 54–55.8 Gy and occasional chemotherapy, particularly for high-risk disease, with unknown outcomes [45, 46]. However, such conventional radiation therapy is associated with progressive neurotoxicity, leading to the degradation of the quality of life. An ongoing phase II trial (NCT01857453) investigates the effects of reduced irradiation compensated by carboplatin- and etoposide-based chemotherapy in newly diagnosed, clinically standard-risk adult patients (ages 18 to 70) (Table 1). The study also aims to assess the relevance of prognostic biomarkers used in the pediatric population for older patients.

### Molecular classifications and subgroup-specific molecular alterations

Given the wide variety of responses to standard of care treatment approaches, the current challenge is to identify patients for whom reduced intervention may boost cure rates while alleviating long-term adverse sequelae or alternatively to identify those who are incurable with paradigm treatments and whom participation in clinical trials would be recommended. Thus, recognizing molecular subgroups provides an opportunity to personalize therapy and avoid over- or under-treatment.

An early genomics study investigating gene expression-based clustering partitioned MB into separate subgroups, each with different histologic, molecular and clinical profiles [47], and the results were corroborated by subsequent analyses with extended sample sizes [12, 48–50]. Four main molecular subgroups were defined [7], and the new subclassification was integrated into the revised WHO system published in 2016 [6]. WNT- and SHH-activated MBs are clearly separable entities from the other subgroups, while no dominant signaling pathway alterations were identified in Group 3 and Group 4 MBs. The latter ones are more related to each other than to the other MBs and therefore appear as non-WNT/non-SHH in the revised WHO classification [6]. The subgroup assignment is still provisory and flexible to further alterations, as Group 3 and 4 MBs encompass a heterogeneous population at both the molecular and clinical levels [13, 49, 51]. Subgroup identity is highly prognostic with markedly different outcomes across the four principal subgroups [11], although survival also strongly depends on histology, presence of metastases, and molecular abnormalities [7, 12, 52, 53].

#### WNT-activated MBs

**Fig 1:**
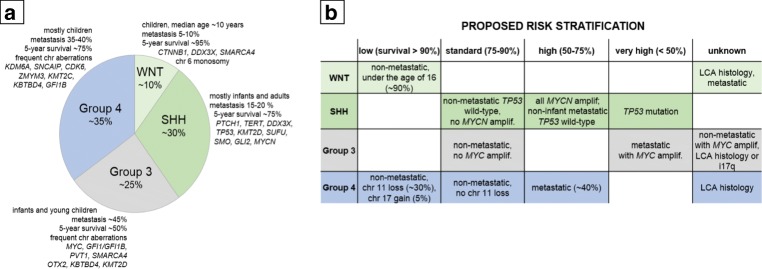
(**a**) The four molecular subgroups of MBs according to the current consensus and (**b**) the proposed risk stratification, based on [2]. WNT-activated (WNT-MB), SHH-activated (SHH-MB), and Group 3 and Group 4 MBs are distinguished by origin, demographics, genetic and molecular alterations, and clinical outcome

WNT-MBs are the least common, accounting for about 10% of all MB patients (Fig. 1). More than 90% of tumors display classic histology, and metastasis is rare with ~ 9% frequency at diagnosis [49, 52]. WNT-MBs are typically absent in infants and are found among older children (median age of 10–12 years), with almost equal ratio of males and females [7, 54]. Prognosis under 16 years of age is usually excellent, with more than 90% 5-year event-free survival [55]. The first European prospective study of MB biomarkers investigating non-metastatic, clinically defined standard-risk tumor samples validated the good prognosis associated with WNT-activated MBs. Higher frequency of relapses were observed in patients older than 16 years of age at diagnosis and in those with delayed radiotherapy, indicating that radiation is an important treatment component [56]. Outcome in adult patients (over 16 years of age) requires further investigations [57, 58]. A recent large-scale study integrating gene expression and methylation data divided WNT-activated MBs into two subtypes: WNT α consists predominantly of children with chromosome 6 monosomy and WNT β represents adults mainly with diploid chromosome 6. The two subtypes harbor similar survival rates, although prognosis in adults is worse and underlying pathway activations are different [58, 59].

Stabilizing mutation of the *CTNNB1* gene is the most common genetic alteration, present in 85–90% of all WNT-activated MBs [8, 60], frequently associated with chromosome 6 monosomy, occurring in about 70–80% of patients [8, 47, 61]. In the large-scale MAGIC study, about 5% of WNT-activated MBs with typical WNT-signatures had neither *CTNNB1* mutations nor chromosome 6 monosomy [62]. Thus, diagnosis solely based on these two events may miss up to 10–15% of WNT-activated MBs [8]. *CTNNB1* mutations frequently occur with mutations of chromatin remodeling genes (*CREBBP*, *TRRAP*, and *MED13*) and/or subunits of the SWI/SNF nucleosome-remodeling complex (*SMARCA4*, *ARID1A*, and *ARID2*) suggesting that besides activation of CTNNB1, aberrant chromatin regulation may also be necessary for WNT-MB [8, 60, 61]. Half of β-catenin mutated WNT-activated MBs harbor concurrent *DDX3X* mutations [61], but these mutations are not subgroup-specific: 10–20% of SHH and 3% of Group 3 tumors also carry *DDX3X* mutations [60]. *DDX3X* participates in multiple cellular processes, including mRNA splicing, export, and translation [63], and its mutations are predicted to increase the proliferation rate of the lower rhombic lip progenitors [60]. *DDX3X* is located on the X chromosome, and not unexpectedly its mutations have a strong male predominance [64]. The relatively frequent *CSNK2B* (wingless signaling pathway) and *EPHA7* (cellular signaling) mutations are additional clinically actionable targets [8]. Although *TP53* mutations are also relatively frequent, these have no prognostic significance in WNT-MBs [8, 61, 65].

The rare germline mutation of *APC* on chromosome 5 as part of the inherited Turcot syndrome contributes to the development of familial WNT-MBs. *APC* mutations are coupled with distinct gene expression signatures, suggesting that *CTNNB1* and *APC* mutations are mutually exclusive and molecularly distinct [8]. Rare germline lesions in anaplastic lymphoma kinase gene (*ALK*) have recently been identified in WNT-activated MBs [66], while other alternative mechanisms, such as mutations of *CDH1*, may promote tumor development [60]. There are no frequent, targetable somatic copy number aberrations in WNT-MBs [8, 62].

#### SHH-activated MBs

SHH-MBs account for ~ 30% of all MBs with an intermediate prognosis (5-year OS of 70%) [49] and are most frequently found in infants and adults, with relatively fewer childhood cases [8, 10, 52]. Age at diagnosis is associated with distinct molecular and clinical categories [57, 67]. The majority of SHH-MBs are nodular/desmoplastic but can also have a classic or LCA histology, the latter especially frequent among children [54, 68]. The nodular desmoplastic subtype predicts increased survival in infants [44, 69, 70].

Over 95% of SHH-MBs contain at least one driver event. However, the types of mutations are highly variable [8, 68]. SHH-MBs have the highest, 14–20% prevalence of destructive germline mutations among all MBs. Li-Fraumeni syndrome is linked to hereditary *TP53* mutations (~ 20% of all SHH-MB), most frequent among children aged 5–16, predisposing to multiple familial cancers and coupled with a low survival [71]. Li-Fraumeni syndrome-associated SHH-MBs have an exceptionally high mutational rate [72], and radiation therapy can accelerate tumor growth [73]. Gorlin syndrome is associated with mutations affecting *PTCH1* and *SUFU* genes of the SHH-pathway [74], present in 21% of all infant SHH-MBs, where radiation also increases the likelihood of secondary, mostly skin cancers [75].

Activating mutations almost permanently involve the SHH-signaling pathway [68], including the mutually exclusive and age-group specific *PTCH1* (~ 43%), *SUFU* (~ 10%), and *SMO* (~ 9%) mutations [8, 60, 68, 72]. *SUFU* and *SFO* mutations are enriched in infants (0–4 years) and adults, respectively, and the presence of *PTCH1* mutations is roughly equal in all age groups [68]. In children, *TP53* mutations are mutually exclusive with *PTCH1* mutations but frequently co-occur with *GLI2* and *MYCN* amplifications, essential regulators of transcription [8]. Loss of chromosome 17p loss is also frequent [68], as are *TP53* mutations that interestingly do not confer a survival disadvantage [72]. *TP53* mutational status separates SHH-MBs with distinct outcomes: tumors with *TP53-*wildtype are more frequent among adults and young children and are linked to favorable prognosis (81% 5-year OS). In contrast, *TP53* mutations are typical among older children and are associated with dismal outcome (40% 5-year OS). *TP53* mutations account for two-thirds of deaths in children older than 5 years. In SHH-MB, more than half of *TP53*-mutantion are germline [65]. Mutations of some genes are almost exclusively specific to the adult subgroup, including *TERT* promoter mutations that drive telomerase activity [76] or recurrent mutations of the PI3K/AKT/mTOR pathway [68]. Additional subgroup-specific molecular alterations affect chromatin modulation, histone acetyltransferase, or nuclear receptor corepressor complexes [77]. High expression often cannot be traced to specific mutations or chromosome aberrations; like in the case of cMET that is among the most frequently hypo- or hypermethylated genes in MBs [78].

#### Group 3 and Group 4 MBs

Group 3 MBs represent approximately 25% of all cases (Fig. 1) with a peak of diagnosis between ages 3 and 5 years and is the deadliest of all MBs, with a 58% 5-year OS in children and a 45% in infants [9, 13, 52]. Male to female ratio is about 2:1 [12]. Group 3 MBs are mostly classical, although 40% harbor LCA histology, assigning most LCA tumors to this subgroup [12]. The aggregation of multiple adverse circumstances, such as young age coupled with metastatic spread (up to 50% of patients), the presence of MYC amplifications, and high rate of LCA histology, all contribute to the poor prognosis. *MYC*-amplified tumors confer an especially short survival with only 20% of patients surviving 5 years post-diagnosis [49, 79]. Absence of metastatic spread does not necessarily reflect good prognosis [67]; thus, standard-risk children with Group 3 disease may face under-treatment [52].

Group 4 is the most prevalent (~ 35%, Fig. 1) and also the least understood subgroup [7, 52] including about 45% of all childhood and 25% of adult cases with an overall intermediate prognosis (5-year overall survival 75–90%) [7]. The prevalence is three times higher in males than in females [7, 52]. Outcome is excellent in patients with chromosome 11 loss where survival exceeds 90% [2]. Nevertheless, 30–40% of Group 4 MB patients are diagnosed with metastases and treated for high-risk disease, with 5-year survival as low as 60% [2, 11, 52]. Adults with Group 4 MBs have a significantly worse prognosis compared to the SHH- or WNT-activated subtypes [58].

Group 3 and Group 4 MBs are genetically heterogeneous, not driven by well-defined, constitutively activated signaling pathways, and rarely have damaging germline mutations in known cancer predisposition genes [71]. Tetraploidy is a recurrent early genetic event, leading to an increased number of large-scale copy number gains [72]. The most frequent cytogenetic alterations in Group 3 MBs include the loss of 17p (55%) along with loss of 16q (42%), 10q (43%), and 9q (21%) and the gain of 17q (in 58% of samples), 7 (39%), and 1q (41%) [52]. Somatic *MYC* (17% in Group 3) and *MYCN* (6% Group 4) amplifications are frequent driver events [62], and high MYC levels are associated with meager outcome [49, 80]. Other recurrent genetic events in Group 3 MBs include *OTX2* and *EZH2* amplifications, *SMARCA4* mutations, *GFI1* enhancer activation, copy number alterations in TGFβ pathway genes, and mutations in Notch signaling genes [81]. Tetraploidy is also present in approximately 40% of Group 4 tumors with unknown prognostic significance [72]. Isochromosome 17q occurs in about 80% of Group 4 tumors, but it does not confer poor prognosis. Other major recurrent genetic events in Group 4 MBs include 8p loss (41%), 10q loss (15%), aberrations of 11p and 18q, gain of chromosome 7 (47%), complete loss of one X chromosome in about 80% of females [49, 52, 67, 82], amplifications of *MYCN* and *CDK6*, *SNCAIP* duplications, and mutations in genes responsible for chromatin modification, such as *KDM6A* [81].

### Emerging risk stratification models

A 2015 consensus conference held in Heidelberg incorporated prognostic molecular markers and suggested a more refined risk stratification for non-infant tumors (ages 3–17). The proposed four categories include low, standard, high, and very high-risk MBs [2] (Fig. 1). The low-risk (survival > 90%) group slots in non-metastatic patients under the age of 16 with WNT-MBs [2], possibly including patients with incomplete resections [13]. Patients over 16 years of age may not be low-risk, and recommendations for patients harboring metastatic disease or LCA histology are unclear. Non-metastatic Group 4 patients with chromosome 11 loss (about one-third) and/or whole gain of chromosome 17 (about 5%) also qualify as low-risk [11]. The standard-risk (survival 75–90%) population includes non-metastatic SHH patients excluding *TP53*-mutated or *MYCN* amplified tumors, non-metastatic Group 3 MBs with no *MYC* amplification, and non-metastatic Group 4 MBs without chromosome 11 loss [11, 51, 65]. The high-risk (50–75% survival) cohort consists of all *MYCN*-amplified SHH-activated MBs (regardless of the metastatic status): *TP53* wild-type, non-infant metastatic SHH-activated and metastatic Group 4 MBs [11]. The very high-risk (< 50% survival) population includes SHH patients with *TP53* mutations and metastatic Group 3 MBs with *MYC* amplifications [2, 11, 65]. The risk evaluation of non-metastatic but *MYC*-amplified Group 3 tumors with an LCA histology or isochromosome 17q or Group 4 MBs with anaplastic histology requires further clarifications [2].

Molecular classification of MBs is constantly evolving, and additional stratification schemes started to emerge based on genome-wide molecular approaches. A large-scale study integrating gene expression and methylation data by similarity network fusion divided each subgroup into further biologically and clinically relevant subtypes. The resulting 12 subtypes (2 in WNT, 4 in SHH, and 3 in both Group 3 and Group 4) are suggested to be different on the molecular, clinical, and prognostic levels [59]. Another study utilizing methylation signatures and unsupervised class discovery distinguished seven robust subtypes, all predictive of clinical outcome. The analysis splits current subgroups, except WNT, into further subtypes: SHH by an age-dependent manner into infant (< 4.3 years) and children cohorts while both Group 3 and Group 4 into high-risk (HR) and low-risk (LR) categories with dramatically different survival rates. Within each subtype, secondary molecular characteristics strongly influenced prognosis and resulted in four different risk categories from favorable to very high-risk. The overall model is suggested to outperform other MB risk stratifications [16]. Novel subtypes with specific genetic signatures were identified in another large-scale study utilizing somatic landscape and epigenetic analyses [8].

The discrepancies across classification schemes stem from diverse patient populations (children only or all ages), types of data utilized, and clustering methods. Still, the emerging differences hold promise for improved stratification and genotype-specific treatment. Nevertheless, well-planned prospective clinical trials are inevitable to reach a consensus across stratification models and to identify appropriate biomarkers.

### Molecular subgroup-specific treatment approaches

#### WNT-activated MBs

WNT-MBs contain a leaky vascular endothelium that disrupts the blood-brain barrier (BBB) integrity. The lack of functional BBB increases penetration of systemic chemotherapy and contributes to the excellent prognosis seen in WNT-MB patients even in the presence of metastases [83]. The Wnt pathway has a crucial role in various developmental processes, including tissue regeneration and bone formation [84]. Targeting the Wnt pathway would interfere with such physiological mechanisms and could also eliminate the advantageous chemosensitivity of WNT-activated MBs. Consequently, targeted therapies against WNT-MBs are not being developed. Instead, current therapy de-escalation for low- or standard-risk WNT-MBs is tested by several clinical trials (Table 1). Trial NCT01878617 utilizes molecular subgroup allocation and clinical risk category at treatment selection and investigates therapy de-escalation for low-risk WNT-activated MBs. Another phase II trial (NCT02724579) studies the effects of reduced CSI and chemotherapy (no vincristine during radiotherapy and reduced-dose maintenance chemotherapy) on average-risk WNT-activated MBs defined by positive nuclear beta-catenin expression (by IHC), *CTNNB1* mutations, and absence of *MYC* and *MYCN* amplifications (by FISH).

The SIOP PNET 5 phase II/III trial (NCT02066220) evaluates the efficacy of risk-dependent treatment adjustments. The aim is to confirm high event-free survival in patients with a low-risk profile defined by non-metastatic MB with total and near-total tumor resection, absence of *MYC* and *MYCN* amplifications and LCA histology, and nuclear beta-catenin immune positivity. Low-risk (WNT-activated) patients receive radiotherapy with a dose of 54 Gy to the primary tumor and 18.0 Gy to the craniospinal axis without carboplatin, followed by six cycles of reduced-intensity maintenance chemotherapy (consisting of three courses of cisplatin, CCNU, and vincristine alternating with three courses of cyclophosphamide and vincristine).

#### SHH-activated MBs

Inhibition of smoothened (SMO) offers avenues for a targeted subgroup-specific treatment strategy for SHH-activated MBs. SMO inhibition blocks downstream signaling, Gli translocation, and activation of Hedgehog target genes [85]. Most adult SHH-MB patients (80%) carry *PTCH1* or *SMO* mutations. Vismodegib (GDC-0449), a small molecule inhibitor of SMO, demonstrated particular efficacy against recurrent/refractory MBs [86, 87]. Nevertheless frequent mutations downstream of SMO, such as mutations affecting *SUFU* or *GLI1*, render most infants and children resistant to targeted vismodegib therapy [68]. Moreover, SMO inhibition hinders normal bone and teeth development in pediatric patients causing growth plate fusions that persist long after therapy cessation [88]. Preclinical efforts seeking targeted therapies against SHH-MBs also focus at epigenetic treatments with BET bromodomain inhibitors, inhibition of the G2/M regulators AURK and PLK, cMET inhibitors, strategies targeting stem-like cells, and interventions utilizing immunotherapies [77].

The currently recruiting phase II (NCT01878617) trial investigates the feasibility and toxicity of oral vismodegib maintenance therapy after conventional chemotherapy for standard-risk or high-risk newly diagnosed MBs in skeletally mature SHH-activated patients (Table 1). For recurrent/refractory SHH-activated MBs, further treatments and drug combinations are available (Table 2). A phase I trial organized by the St. Jude Children’s Research Hospital (NCT03434262) investigates subgroup-specific doublet combinations including the CDK4/6 inhibitor ribociclib and SMO inhibitor sonidegib (LDE225) for adult SHH-activated patients, with copy number loss of 9q or *PTCH1* mutations and ribociclib and MEK-inhibitor trametinib for WNT- and SHH-activated MBs.

**Table 2:** Ongoing clinical trials currently recruiting patients with recurrent/refractory/progressive medulloblastomas

ID	Phase	Treatment	Subgroup	Eligible age cohort	Estimated enrollment	Estimated primary completion date	Responsible party
NCT00089245	I	Intrathecal radioimmunotherapy using I-8H9	8H9 reactive malignancy confirmed by IHC	Child, adult, older adults	120	2018	Y-mAbs Therapeutics
NCT02502708	I	Indoximod, an inhibitor of the immune “checkpoint” pathway indoleamine 2,3-dioxygenase (IDO), in combination with temozolomide		3 years to 21 years	115	2019	NewLink Genetics Corporation
NCT02684071	II	Methotrexate and chemotherapy (topotecan and cyclophosphamide)		up to 21 years	10	2019	Ziad Khatib, Nicklaus Children’s Hospital f/k/a Miami Children’s Hospital
NCT02905110	Early phase I	Simultaneous methotrexate/etoposide infusion	Posterior fossa tumors	1 year to 80 years	10	2020	David Ilan Sandberg, The University of Texas Health Science Center, Houston
NCT01661400	Early phase I	Metronomic cyclophosphamide or thalidomide		6 months to 21 years	12	2020	Washington University School of Medicine
NCT02962167	I	Modified measles virus (MV-NIS)		12 months to 39 years	46	2020	Sabine Mueller, MD, PhD, University of California, San Francisco
NCT03043391	I	Oncolytic poliovirus (PV) immunotherapy with PVSRIPO		12 years to 21 years	12	2020	Istari Oncology, Inc.
NCT02271711	I	Autologous ex vivo-expanded NK cells		up to 21 years	24	2020	MD Anderson Cancer Center
NCT02359565	I	Pembrolizumab		1 year to 29 years	110	2020	National Cancer Institute (NCI)
NCT03389802	I	APX005M, a humanized IgG1κ mAb that binds to CD40		1 year to 21 years	45	2020	Pediatric Brain Tumor Consortium
NCT03299309	I	Cytomegalovirus (CMV)-specific peptide vaccine (PEP-CMV)		3 years to 35 years	30	2020	Gary Archer Ph.D., Duke University
NCT03734913	I	ZSP1602, a SMO protein inhibitor		18 years to 75 years	65	2020	Guangdong Zhongsheng Pharmaceutical Co., Ltd.
NCT03598244	I	Savolitinib, a small molecule inhibitor of c-Met		6 years to 21 years	36	2020	National Cancer Institute (NCI)
NCT03387020	I	Ribociclib (inhibitor of cyclin D1/CDK4 and CDK6) in combination with everolimus		1 year to 21 years	45	2020	Pediatric Brain Tumor Consortium
NCT03173950	II	Nivolumab		18 years to 99 years	180	2020	National Institutes of Health Clinical Center (CC) (National Cancer Institute (NCI))
NCT03273712	II	90Y-DOTA-tyr3-Octreotide for somatostatin receptor positive tumors	Tumors expressing somatostatin receptors	6 months to 90 years	46	2020	Sue O’Dorisio, University of Iowa
NCT02644291	I	Mebendazole, an antihelmintic agent		1 year to 21 years	21	2021	Sidney Kimmel Comprehensive Cancer Center at Johns Hopkins
NCT03500991	I	HER2-Specific CAR T-cell locoregional immunotherapy		1 year to 26 years	36	2021	Julie Park, Seattle Children’s Hospital
NCT02095132	I/II	Adavosertib (MK-1775), a small molecule inhibitor of the tyrosine kinase WEE1 and irinotecan hydrochloride		1 year to 21 years	154	2021	National Cancer Institute (NCI)
NCT01356290	II	Multidrug antiangiogenic approach		up to 19 years	40	2021	Andreas Peyrl, Medical University of Vienna
NCT03911388	I	G207, an oncolytic herpes simplex virus-1 (HSV)		3 years to 18 years	15	2022	Gregory K. Friedman, MD, University of Alabama at Birmingham
NCT03638167	I	EGFR806-specific CAR T-cell locoregional immunotherapy		1 year to 26 years	36	2022	Julie Park, Seattle Children’s Hospital
NCT03893487	Early phase I	Fimepinostat (CUDC-907), a small molecule inhibitor of histone deacetylase and PI3 kinase		3 years to 39 years	30	2022	Sabine Mueller, MD, PhD, University of California, San Francisco
NCT03709680	I	Palbociclib in combination with temozolomide and irinotecan		2 years to 20 years	100	2022	Pfizer
NCT03904862	I/II	CX-4945 (silmitasertib sodium), small molecule inhibitor of casein kinase II (CK2)	SHH	3 years and older	60	2022	Pediatric Brain Tumor Consortium
NCT02748135	I/II	TB-403 (humanized monoclonal antibody against placental growth factor (PlGF)		6 months to 18 years	36	2022	Oncurious NV
NCT03936465	I	BMS-986158, a bromodomain (BRD) and extra-terminal domain (BET) inhibitor	Predicted increased sensitivity to BET inhibition: MYC or MYCN amplification or high copy number gain, MYC or MYCN translocation, BRD4 or BRD3 translocation	1 year to 21 years	34	2022	Steven DuBois, Dana-Farber Cancer Institute
NCT02650401	I/II	Entrectinib (RXDX-101), a TRKA/B/C, ROS1, and ALK inhibitor	CNS tumors harboring- NTRK1/2/3, ROS1, ALK molecular alterations, including gene fusions	up to 22 years	65	2023	Hoffmann-La Roche
NCT04049669	II	Indoximod in combination with radiation and chemotherapy		3 to 21 years	140	2024	Theodore S. Johnson, Augusta University
NCT03210714	II	JNJ-42756493 (erdafitinib), an oral pan-FGFR inhibitor	Mutations in the FGFR1/2/3/4 pathway	12 months to 21 years	49	2024	National Cancer Institute (NCI)
NCT03213678	II	Ly3023414 (samotolisib), a PI3K/mTOR inhibitor	TSC loss of function mutations, PI3K/MTOR activating mutations	12 months to 21 years	144	2024	National Cancer Institute (NCI)
NCT03213704	II	LOXO-101 (larotrectinib)	NTRK fusions	12 months to 21 years	49	2024	National Cancer Institute (NCI)
NCT03213665	II	Tazemetostat, a small molecule EZH2 inhibitor	EZH2, SMARCB1, or SMARCA4 gene mutations	12 months to 21 years	49	2024	National Cancer Institute (NCI)
NCT03233204	II	Olaparib	Defects in deoxyribonucleic acid (DNA) damage repair genes	12 months to 21 years	49	2024	National Cancer Institute (NCI)
NCT04023669	I	Prexasertib (LY2606368), a molecularly targeted CHK1/2 inhibitor	Group3/Group4; SHH; indeterminate	1 year to 24 years	100	2025	St. Jude Children’s Research Hospital
NCT03434262	I	Ribociclib and gemcitabine	Group3/Group4	1 year to 39 years	108	2025	St. Jude Children’s Research Hospital
		Ribociclib and trametinib	SHH/WNT	1 year to 39 years	108	2025	St. Jude Children’s Research Hospital
		Ribociclib and sonidegib	SHH	1 year to 39 years	108	2025	St. Jude Children’s Research Hospital
NCT03526250	II	Palbociclib	Rb positive, or mutations in cell cycle genes	12 months to 21 years	49	2025	National Cancer Institute (NCI)
NCT03155620	II	Treatment directed by mutation carrier screening	Based on results of molecular screening	12 months to 21 years	1000	2027	National Cancer Institute (NCI)

The Pediatric Brain Tumor Consortium Phase I/ II trial NCT03904862 evaluates the effects of CX-4945, silmitasertib sodium, a small molecule inhibitor of Casein kinase II (CK2) in skeletally immature (phase I) and skeletally mature (phase II), SHH-activated recurrent/refractory MB patients, where subgroup identity is confirmed by a CLIA certified methylation-based assay. The St. Jude ELIOT (NCT04023669) phase I trial evaluates prexasertib (LY2606368), a targeted CHK1/2 inhibitor in combination with cyclophosphamide for recurrent/refractory SHH-activated or Group 3/Group 4 MBs. Another trial, NCT03734913, explores ZSP1602, a SMO protein inhibitor, designed for adult patients with advanced MBs, regardless of SMO or Gli1 alteration status and molecular subgroup identity.

#### Group 3/Group 4 MBs

The limited understanding of tumorigenesis mechanisms hampers the development of targeted treatment strategies for Group 3 and Group 4 MBs. For newly diagnosed patients, the NCT01878617 trial offers risk-adapted treatment strategy: intermediate- and high-risk patients are exposed to new chemotherapy agents (pemetrexed and gemcitabine) after standard chemotherapy and risk-adapted radiotherapy (Table 1).

For recurrent and refractory MB patients, two trials offer treatments based on molecular subgrouping (Table 2). In NCT04023669, prexasertib (LY2606368), a targeted CHK1/2 inhibitor, is administered in combination with cyclophosphamide for Group 3, Group 4, and SHH-activated MBs or in combination with gemcitabine for Group 3/Group 4 MBs. The trial NCT03434262 investigates the combination of the cyclin-dependent kinase inhibitor ribociclib and gemcitabine for Group 3/Group 4 MBs.

In Group 3 MBs, preclinical studies focus at the inhibition of PI3K and mTOR signaling pathways, evaluate the synergistic activity between histone deacetylase inhibitors and PI3K inhibitors, assess the efficacy of CDK, PRKDC or BET bromodomain inhibitors, and test the effectiveness of anti-vascularization therapies [81]. Such experiments are no longer confined to preclinical model systems, and numerous early phase clinical trials started to explore these promising avenues in recurrent/refractory MBs, although therapeutic approaches rarely reflect the existing MB molecular classifications (Table 2).

### Refractory, recurrent, and progressive MBs

Metastases and recurrence are the major barriers to therapeutic success, and disease recurrence is responsible for 95% of MB-associated deaths [89]. Patients who relapse after receiving upfront radiotherapy rarely survive (salvage rate < 10%) despite the multitude of treatment options [90, 91]. Most SHH tumors recur in the tumor bed, suggesting that focusing therapies at the posterior fossa could be most profitable. By contrast, Group 3 and Group 4 MBs develop distant metastases irrespectively of the type of therapy administered at diagnosis [92]. Interestingly, Group 4 patients recur later compared to the other subtypes and survive longer after recurrence. Although metastases are rare in WNT-activated MBs, recurrence may occur locally with or without systemic dissemination [91, 92].

Molecular subgroup affiliation remains stable across samples collected at diagnosis and relapse [92, 93]. However, substantial genetic divergence occurs [94–96]. About two-thirds of relapses acquire high-risk features, including *TP53-MYC* gene family interactions. The presence of *TP53-MYC* interactions is associated with rapid disease progression [95]. Whole-genome sequencing identified significant increase in somatic mutational burden post-therapy: mutations of *TP53* and other genes of the *TP53* pathway, such as *DYNC1H1* and losses of chr14q, occurred frequently, predominantly in recurrences of SHH-MBs [94]. Putative drug targets present at diagnosis were retained in only 44% of patients post-therapy, providing an explanation why most targeted therapies fail in MB [94].

The difficulty to manage recurrent/refractory/progressive MBs is reflected in the number of clinical trials dedicated to this population: about 80% of currently recruiting studies focuses on these heavily pretreated patients, although two-thirds of the identified trials are still in early phase (Table 2).

#### Targeted therapies of CNS tumors irrespective of molecular subgrouping

Several trials are not explicit to MBs and recruit patients with a range of advanced solid tumors including various neoplasm of the CNS. Such is the early phase I NCT03893487 trial exploring the ability of fimepinostat (CUDC-907), a small molecule inhibitor of histone deacetylase and PI3 kinase, to penetrate the blood brain barrier (BBB) of children and young adults with various CNS tumors, including recurrent MBs of any molecular subtypes.

The efficacy of cyclin-dependent kinase (CDK) inhibitors in treating hormone receptor positive, HER2-negative advanced breast cancer boosted the interest toward their further use in other tissue types. There are currently 103 active and recruiting clinical trials as listed at clinicaltrial.gov evaluating palbociclib alone in various tissue types including primary CNS tumors. The maximum tolerated dose of palbociclib (CDK4/6 inhibitor) combined with chemotherapy (irinotecan and temozolomide) is evaluated in the NCT0370968 phase I trial in children with solid and CNS tumors. A combined ribociclib (cyclin D1/CDK4 and CDK6 inhibitor) and everolimus treatment is utilized in the NCT03387020 phase I trial in children with recurrent/refractory primary CNS tumors, including MBs, with an intact RB1 protein.

Entrectinib (RXDX-101), a TRKA/B/C, ROS1, and ALK inhibitor, is being studied in a phase I/II trial (NCT02650401) recruiting children and young adults with recurrent/refractory CNS tumors harboring NTRK1/2/3, ROS1, and ALK molecular alterations, as confirmed by a CLIA-approved lab. A National Cancer Institute (NCI) sponsored phase I trial experiments with savolitinib, a small molecule inhibitor of c-Met (NCT03598244) in primary brain tumors including recurrent/refractory MBs with MET pathway activations, defined as MET mutations/amplifications/fusions, HGF amplifications, and gain of chromosome 7. Another NCI sponsored phase I/II study (NCT02095132) evaluates the activity of adavosertib (MK-1775), a small molecule inhibitor of the tyrosine kinase WEE1, combined with irinotecan hydrochloride in relapsed or refractory solid malignancies including CNS tumors. The study also aims to evaluate predictive biomarkers of adavosertib sensitivity, such as the expression of *MYC*, *MYCN*, *EZH2*, *H2AX*, and phosphorylated *WEE1*. The NCT03936465 trial evaluates the safety and dosing of the investigational drug BMS-986158, a bromodomain and BET inhibitor in pediatric solid tumors with molecular features predicted to increase sensitivity to BMS-986158, consisting of *MYC* and *MYCN* translocations and amplifications and *BRD4* or *BRD3* translocations. Additional ongoing early phase trials evaluating the safety and tolerability of novel treatment strategies are listed in Table 2.

#### Targeted therapies irrespective of the tumor or tissue type

There is an increasing tendency in oncology to explore the efficacy and safety of molecularly targeted agents for sample-specific genetic alterations, irrespectively of the tumor or tissue type (Table 2). Such is the NCI-COG Pediatric MATCH (Molecular Analysis for Therapy Choice) trial (NCT03155620) that evaluates experimental treatments targeting selected molecular alterations in children and adolescents with advanced solid tumors. Drug selection is based on the results of molecular screening with genome sequencing, with at least 20 patients in each treatment arm. Six phase II clinical trials concerning recurrent/refractory MBs are currently recruiting within the program: patients harboring mutations in the FGFR1/2/3/4 pathway may receive erdafitinib (JNJ-42756493), an oral pan-FGFR inhibitor (NCT03210714); MBs with TSC loss of function mutations or PI3K/mTOR activating mutations are treated with samotolisib (Ly3023414), a PI3K/mTOR inhibitor (NCT03213678); actionable NTRK fusions are treated with larotrectinib (LOXO-101) (NCT03213704); mutations in RB or in cell cycle genes are targeted by palbociclib (NCT03526250); tumors with defects in DNA damage repair genes are targeted by olaparib (NCT03233204); and EZH2, SMARCB1, or SMARCA4 gene mutations are treated with tazemetostat, a small molecule EZH2 inhibitor.

### Immunotherapies for advanced disease

The expanding number of new trials investigating immunotherapies reflects recent tendencies in oncology. Currently, the number of phase I studies utilizing an immunotherapy-based and a targeted approach is equivalent (Table 2).

#### Immune checkpoint inhibitors (ICIs)

Cancer cells upregulate immune checkpoints to escape T-cell-mediated immune response [97]. Groundbreaking application of immune checkpoint inhibitors in numerous solid tumors, including metastatic melanoma and renal cancer [98, 99], generated interest in their applicability in CNS cancers. The approach is especially attractive for patients with advanced disease and limited options for therapy. The success of using ICIs is limited so far – of note, most studies are retrospective and observational, with a limited sample size and unselected patient population. The anti-PD-1 agent pembrolizumab did not show clinical or histologic efficacy in pretreated patients with progressive brain tumors [100], and PD-1 inhibition with nivolumab provided limited benefits for adult patients with recurrent high-grade gliomas [101]. A retrospective review identified survival advantage of nivolumab among pediatric patients with recurrent/refractory brain tumors, especially among PD-L1-positive patients with elevated tumor mutation burden [102]. A phase I clinical trial reported the safety of ipilimumab in pediatric patients with advanced solid tumors, mostly melanomas, with increased overall survival among those with immune-related toxicities [103].

Currently, two clinical trials are investigating blockade of classical inhibitory checkpoint pathways in MBs (Table 2). The goal of the NCT02359565 phase I trial is to assess the safety and efficacy of pembrolizumab in children and young adults with recurrent/refractory/progressive brain tumors including MBs. NCT03173950, a phase II trial, evaluates the effects of biweekly nivolumab administration on objective response rate and progression-free survival of adult patients with rare CNS tumors including MBs.

The B7 homolog 3 (B7-H3), also known as CD276, is also an important checkpoint molecule with an inhibitory function on T-cell activation [104]. The NCT00089245 phase I study evaluates the effectiveness of intrathecal radioimmunotherapy using radioactive iodine labeled antibody I-8H9 against B7-H3, which, after internalization, induces cell death.

In addition to CTLA4, PD-1, and PD-L1 inhibitors, the next generation of immune-checkpoint inhibitors is focusing on other immune pathways. Molecules of the co-stimulatory pathways augment the immunological response against tumor cells. Such is the surface receptor CD40, a TNF receptor family member expressed by APCs and B cells, that stimulates cytokine expression and activation of T cells and induces tumor cell death [105]. CD40 has also direct cytotoxic effects on CD40-positive tumor cells. APX005M, a humanized IgG1κ monoclonal antibody agonist of CD40 is being evaluated in a phase I pediatric trial (NCT03389802) in patients with recurrent/refractory primary malignant CNS tumors.

Indoleamine 2,3-dioxygenase (IDO) is a tryptophan-degrading enzyme, and the produced active metabolites increase T-reg activity while decrease the activity of CD8 T cells, leading to an immunosuppressed tumor microenvironment and enhanced immune escape. IDO is frequently overexpressed in various tumor types [106] and is also associated with poor outcome and resistance to chemotherapy [107]. Indoximod, an inhibitor of the IDO pathway, is being studied with concomitant use of temozolomide in a phase I pediatric trial (NCT02502708) with an estimated completion date in December 2019. Additionally, in an NCI-funded phase II trial (NCT04049669), indoximod complemented with oral temozolomide is given to children and young adults both eligible and not-eligible for partial re-irradiation with progressive brain cancers.

#### Oncolytic viruses

Cancer therapy utilizing oncolytic viruses (OVs) has several advantages over traditional approaches: OVs replicate selectively only in cancerous but not in normal cells, and after infecting a few cells, OVs quickly spread through the entire tumor. The antitumor response is based on a dual mechanism of action: first, OVs induce direct lysis of tumor cells, and second, as a result, the release of neoantigens to the tumor microenvironment triggers activation of the immune system, facilitating tumor eradication [108]. Based on promising results from preclinical studies, several types of viruses are being investigated in the treatment of pediatric brain tumors [109].

A single injection of a genetically engineered herpes simplex virus (HSV) variant, rRp450 to orthotopic mouse xenografts representing Group 3 and 4 MB cells, prolonged survival and had the ability to bioactivate the prodrug cyclophosphamide within the tumor microenvironment [110]. Another set of engineered variants of the HSV, G207, and M002 displayed antitumor activity and prolonged survival of mice bearing xenografts originating from the most aggressive Group 3 or 4 pediatric MB subgroups [111]. The safety of the G207 HSV-variant augmented with radiation has already been determined in a phase I trials in adult patients with recurrent high-grade gliomas [112]. The primary HSV entry receptor nectin-1 (CD111), an adhesion molecule, is expressed in significantly higher amounts in pediatric tumor cells compared to adults and displays sensitivity to the virus in vitro, requiring a substantially lower dose [113]. The results suggest that HSV treatment could be sufficiently effective for treating pediatric brain cancers. Accordingly, a currently recruiting phase I trial (NCT03911388) evaluates the effects of engineered HSV G207 alone or in combination with a single low-dose radiation in children with recurrent/refractory brain tumors, including recurrent MBs.

Measles virus (MV) is a negative strand RNA virus that induces the fusion of neighboring cells leading to the development of giant, metabolically active, multinucleated aggregates, called syncytia, which contribute to cell death [114]. An MV that expresses thyroidal sodium iodide symporter (NIS) has been engineered to monitor viral replication – the expression of NIS also enhanced therapeutic efficacy [114]. There is currently a phase I study (NCT02962167) that aims to evaluate safety and dosing of MV-NIS for the treatment of children and young adults with recurrent MBs or recurrent atypical teratoid rhabdoid tumors (ATRT). For locally recurrent disease, the virus is introduced directly to the tumor bed. For disseminated MB, MV-NIS is injected to the subarachnoid space through lumbar puncture.

Targeted elimination may also be achieved through the highly attenuated polio/rhinovirus recombinant PVSRIPO that recognizes the poliovirus receptor CD155 widely expressed by both cancerous cells and components of the tumor microenvironment [115]. A phase I dose-finding and toxicity study on adults with recurrent supratentorial WHO grade IV malignant gliomas treated by intratumoral delivery of the recombinant polio/rhinovirus chimera identified better survival outcome compared to historical controls [116]. Following a similar approach, a current phase Ib pediatric study (NCT03043391) investigates the safety and dosing of PVSRIPO by convection-enhanced delivery (CED) using an intracerebral catheter in WHO grade III, grade IV gliomas, and other brain tumors including recurrent MBs.

Glioblastomas are lethal brain tumors with no specific therapy. Cytomegalovirus (CMV) antigens are almost universally present and homogeneously expressed in glioblastomas compared to normal brain tissues. Thus, CMV presence provides an excellent opportunity to subvert these immunogenic viral proteins to be tumor-specific targets [117]. A cost effective, two-component CMV-specific multi-epitope peptide vaccine (PEP-CMV) has been created including a synthetic long peptide of 26 amino acid residues from human pp65 and a neutralizing antibody epitope from human CMV glycoprotein B. The phase I PRiME clinical trial (NCT03299309) evaluates the safety of PEP-CMV in pediatric and young adult patients with recurrent MBs and malignant gliomas. Vaccination is preceded by temozolomide treatment to augment immunogenicity, and patients older than 18 years of age receive an intradermal tetanus vaccine booster to precondition the immune system.

#### CAR-T therapy

In the CAR-T therapy, genetically engineered T-cell expressing artificial chimeric antigen receptors (CAR) with both antigen-binding and T-cell activating properties are utilized. CAR-T therapy resulted in breakthroughs in treating hematological malignancies, but solid tumors pose a wide array of challenges, such as finding tumor-associated antigens enriched in tumors but not expressed in normal tissues [118]. HER2 is frequently overexpressed in MBs, and intraventricular administration of autologous HER2-CAR T cells effectively cleared orthotopically implanted MBs in the posterior fossa originating from Daoy and D283 tumor cells in a preclinical mouse MB model [119]. In the BrainChild-01 phase I trial (NCT03500991) of Seattle Children’s Hospital, autologous CD4+ and CD8+ T cells lentivirally transduced to express HER2-specific chimeric antigen receptor and EGFRt (a truncated form of the human epidermal growth factor receptor) are investigated. Engineered T cells are delivered to the tumor resection cavity or the ventricular system of recurrent/refractory MB patients with HER2-positive disease. By this delivery method, T cells do not need to penetrate the blood-brain barrier, and this can presumably provide higher efficiency compared to intravenous infusion. In another Seattle Children’s Hospital phase I trial (NCT03638167), transduced CAR-T cells expressing the EGFR806 specific-CAR and EGFRt are infused into pediatric and young adult patients with histologically diagnosed EGFR positive recurrent/refractory CNS tumors.

#### Natural killer cells

Natural killer (NK) cells play a major role in the immune defense against malignant disease by recognizing a broad spectrum of tumor cells without specific antigen identification [120]. NK cells are controlled by both activating and inhibitory receptors, and MB cell lines express specific ligands that are able to trigger NK activation, enabling the lysis of MB cell lines in vitro regardless of the presence or absence of the stem cell marker CD133 [121]. However, complete elimination is unlike due to inhibitory signals generated by the tumor cells. Several studies investigate ex vivo propagated, transduced, or otherwise modified NK cells, such as cells expressing dominant-negative TGF-β receptor II (DNRII) [122]. In a currently recruiting phase I clinical trial by the MD Anderson Cancer Center (NCT02271711), ex vivo propagated autologous NK cells with artificial antigen-presenting cells (aAPC) are being administered directly to the brain of pediatric patients with recurrent/refractory MBs.

## Conclusions

Recent advances in MB research have expanded our understanding of MB pathogenesis and expanded the list of potential biomarkers with prognostic significance. Prospective trials are needed to confirm the validity of molecularly grounded stratifications, eventually offering more precise treatment strategies. The ongoing transition toward molecularly guided clinical interventions is slow, especially for newly diagnosed MB patients. Clinical trials aim to fine-tune invasive radio- and chemotherapies to reduce intervention-related morbidity in low- or standard-risk patients, with a particular focus on young children and rare MBs affecting adults. Studies recruiting newly diagnosed patients exploit molecular markers to some extent, especially to distinguish WNT-activated MBs utilizing mandatory testing for *CTNNB1* mutational status or IHC positivity. Evaluation of the presence of *MYC* or *MYCN* amplifications to identify high-risk patients also became standard procedure. Nevertheless, incorporation of a full molecular analysis for newly diagnosed patients utilizing both the clinical risk and the tumor’s molecular subgroup allocation for therapy selection is limited to a single trial (NCT01878617).

It is especially vital to unravel novel, druggable pathways for subgroups with frequent metastases and low rates of recurrent molecular alterations, such as Group 3 and Group 4 MBs. For high-risk patients, clinical trials evaluate the effects of therapy intensification and novel chemotherapy drugs. Emergence of innovative concepts, such as integration of proteomic approaches into existing models of tumorigenesis, may expand translational opportunities [123].

The proportion of targeted and immunotherapies in recurrent/refractory MBs is increasing compared to cytotoxic strategies. Several trials consider molecular subgrouping, and novel targeted therapies are being explored irrespectively of the tumor or tissue type. Immune therapies utilize a wide range of treatment options, from immune checkpoint inhibitors, CAR-T therapies, and NK-cells to oncolytic viruses. While most trials are in an early phase, there is hope for therapeutic breakthroughs in historically difficult to treat recurrent/refractory MBs in the next decade.
